# Morphology-controlled construction of hierarchical hollow hybrid SnO_2_@TiO_2_ nanocapsules with outstanding lithium storage

**DOI:** 10.1038/srep15252

**Published:** 2015-10-20

**Authors:** Linzong Zhou, Hong Guo, Tingting Li, Weiwei Chen, Lixiang Liu, Jinli Qiao, Jiujun Zhang

**Affiliations:** 1 School of Chemistry Science and Engineering, Yunnan University, Kunming 650091,Yunnan, China; 2 School of geographical science and tourism management, Chuxiong Normal University, Chuxiong 675000, Yunnan, China; 3 College of Environmental Science and Engineering, Donghua University, Shanghai 201620, China; 4 Department of Chemical Engineering, E6-2006, University of Waterloo, Waterloo, ON, N2L 3G1, Canada

## Abstract

A novel synthesis containing microwave-assisted HCl etching reaction and precipitating reaction is employed to prepare hierarchical hollow SnO_2_@TiO_2_ nanocapsules for anode materials of Li-ion batteries. The intrinsic hollow nanostructure can shorten the lengths for both ionic and electronic transport, enlarge the electrode surface areas, and improving accommodation of the anode volume change during Li insertion/extraction cycling. The hybrid multi-elements in this material allow the volume change to take place in a stepwise manner during electrochemical cycling. In particular, the coating of TiO_2_ onto SnO_2_ can enhance the electronic conductivity of hollow SnO_2_ electrode. As a result, the as-prepared SnO_2_@TiO_2_ nanocapsule electrode exhibits a stably reversible capacity of 770 mA hg^−1^ at 1 C, and the capacity retention can keep over 96.1% after 200 cycles even at high current rates. This approach may shed light on a new avenue for the fast synthesis of hierarchical hollow nanocapsule functional materials for energy storage, catalyst and other new applications.

The hollow micro/nanoscale metal oxides are of great interests because of their high specific surface area, well-defined interior voids, as well as the he accommodation of volume change compared to its solid counterparts with the same size^1^,^2^,^3^,^4^,^5^,^6^,^7^,^8^,^9^. Due to these advanced properties, their potential applications are very promising in dye-sensitized solar cells, microreactors, sensors, catalysis, and electrochemical storage systems^7^,^8^,^9^,^10^,^11^,^12^,^13^,^14^. Particularly, as reported, the novel hollow mixed metal oxides containing multi-functional component could exhibit much enhanced performance in the field of environmental and energy science when compared to those with single component hollow structures^15^,^16^,^17^,^18^,^19^. For example, there have been studies demonstrating that hollow TiO_2_ with different morphologies and sizes could have both good catalytic and electrochemical properties^20^,^21^. Our previously prepared yolk-shell structured Pd@CeO_2_ and Ag@TiO_2_ exhibited excellent performance as applied in Li-ion storage, as well as photocatalysis and catalysis^22^. Lou and co-workers synthesized hollow metal sulfides microcubes with excellent Li storage capacity^23^,^24^. Lately, the novel hybrid hollow materials concluding multiple compositions or multiple layers of shells have exhibited desirable performance in target applications and enhanced structural virtues compared with single component ones^16^,^18^,^19^. In this regard, the hollow porous hybrid micro/nano-structures have exhibited considerable application in a wide range of fields regardless of whether fundamental research or practical uses due to their unique characters. However, utilizing conventional template routes to synthesize hollow materials normally can only get relatively simple configurations and should also need long time to remove the template, such as self-assembly of nanostructural cuprous oxide^25^, bio-templates^26^, and carbon^27^,^28^. To address these issues, the effect approaches to fabricate hierarchically hollow hybrid nano/micro-materials rationally are still the key issue, and it is important to obtain these materials through more general and fast process.

SnO_2_ have been considered to be one of the most promising key functional materials for a great variety of practical applications. Most recently, the more intensive attempts have been made to obtain the maximum performance using SnO_2_-based compounds as anode materials because of its high lithium storage of 782 mA hg^−1^. However, SnO_2_-based electrodes endure serious mechanical disintegration because of the drastic volumetric changes in the process of electrochemical reaction, which leads to its capacity deteriorated rapidly. To deal with this problem, it should be allowing the electrochemical reaction to be proceeded in a hybrid matrix of systems such as carbonaceous materials and oxides^12^,^23^,^24^. So, the confining matrix containing active or inactive components towards Li may result in that the volume change occurs in a step-wise manner instead of at a fixed potential. Therefore, the unreacted phase will buffer the strain yielded by reacted component. On the other hand, the electronic conductivity can be enhanced and the SnO_2_-based material with rich redox reactions can be rendered by the coupling of two metal species. For example, Whittingham and coworkers have analyzed the recent commercial SONY tin-based anode, which is to be basically composed of SnCo nanoparticles coated with graphitic carbon^29^. Using nano/micro-particles with various morphologies is another important way to improve the performance of SnO_2_-based electrodes such as nanowall, nanotube, nanobowl, nanocone, and nanosheet, of which the hollow materials have shown particular interest for the reversible lithium ion storage, because they exhibit good toleration for volume change and can short Li^+^ diffusion during cycling. Though these strategies are effective, each design procedure alone always results in limited increase in the electrochemical properties. As a result, the development of a facile, controllable and scalable fabrication of hierarchically hybrid SnO_2_-based materials with good cycling ability and high capacity is still highly desirable for LIBs. It has been reported by Guo *et al*.^30^ that TiO_2_ nanocoating can effectively enhance the electrical conductivity and rate capacity of electrode. However, the researches on the fast synthesis of hierarchically hollow SnO_2_@TiO_2_ nanocapsules are quite rare compared with current methods that produced nanospheres.

Herein, we chose a TiO_2_-SnO_2_ composite material to elaborate our concept and design a facile method to fabricate hollow hierarchical SnO_2_@TiO_2_ nanocapsules as illustrated in Fig. 1. The as-prepared olivary α-Fe_2_O_3_ is hired as the template, and then Sn ions are precipitated in the templates. Subsequently, the hollow SnO_2_ nanocapsules are obtained by microwave-assisted HCl etching process. Finally, titanium ions are used to self-gathering around the surface of SnO_2_ and the thermal treatment facilitates the products of hierarchical hollow TiO_2_@SnO_2_ nanocapsules. The two active components of SnO_2_ and TiO_2_ can realize the high capacity feature and also make the volume change of electrode takes place in a stepwise manner because of the different lithiation potentials of the two active components. So, a stable cycling performance will be obtained. Moreover, our hierarchical hollow structures possess a stable frame without destructive effect during the process of template removal and a relatively high surface area compared with other normal methods synthesized nano metal oxides electrodes. The former can accommodate large volume change, while the latter factor can expand the contact area between Li ions and active components during electrochemical reaction. Moreover, the hollow structures can provide efficient passageways for mass transport and short the length of ionic/electronic diffusion. It is, therefore, that the good cycling performance and high rate capacity can be expected.

## Experimental Section

### Materials and Methods

#### Preparation of monodisperse Fe_2_O_3_ solid particles with different shapes

All the reagents are analytically pure. In a typical synthesis, a NaOH solution (90 mL, 6.0 M) was added to 100 mL of well stirred 2.0 M FeCl_3_ in a 250 mL Pyrex bottle over 5 min, followed by the addition of Na_2_SO_4_ solution (10 mL, 0.60 M), and the agitation was continued for an additional 10 min. The tightly stoppering bottle to promote the synthesis of Fe(OH)_3_ gel, which is mainly of 0.9 M Fe(OH)_3_, 0.1 M Fe^3+^ and 0.03 M SO_4_^2-^, was placed in laboratory furnace preheated to 100 °C and the gel was aged for 8 days. After the treatment, red products were collected by filtration, washed three times with deionized water and ethanol before drying at 50 °C overnight.

#### Preparation of Solid α-Fe_2_O_3_@SnO_2_ nanocapsules and hollow SnO_2_ nanocapsules

The SnCl_4_ powder as Sn source was dissolved in deionized water to form 0.25 M SnCl_4_ solution. For a typical SnO_2_ coating, monodisperse α-Fe_2_O_3_ particles (0.5 g) with capsule shapes were first dispersed by ultrasonication in a mixture consisting of 30 mL of ethanol, followed by addition of SnCl_4_ solution (8 mL, 0.25 M) after being aged for 0.5 h. The suspension was poured into a 50 mL Teflon-lined stainless-steel autoclave after ultrasonication for 20 min, an then they are heated in an air-flow electric oven at 160 °C for 4 h. After the autoclave cooled down naturally, the as-product was filtrated, and then washed three times with deionized water and ethanol before vacuum-drying at 80 °C for 10 h. Finally, the hollow SnO_2_ nanocapsules were obtained by microwave-assisted immersing with a microwave power of 50 W in a dilute HCl solution (0.5 M) at 100 °C for the needing time.

#### Synthesis of hierarchically hollow SnO_2_@TiO_2_ nanocapsules

Under the ultrasonication, the solid α-Fe_2_O_3_@SnO_2_ nanocapsules (0.5 g) were first dispersed in a mixture consisting of 30 mL of ethanol for 30 min, and then 10 mL of TiOSO_4_ solution (0.1 M) as Ti source was added into the above solution. Subsequently, ultrasonication for another 20 min, the solution was poured into a 50 mL Teflon-lined stainless steel autoclave and heated in an air-flow electric oven at 80 °C for 2 h. The products were collected and washed before vacuum drying at 50 °C for 10 h. The products were obtained by annealed in Air atmosphere at 450 °C for 4 h with a slow ramp rate of 1 °C min^−1^ to make the SnO_2_@TiO_2_ nanocapsules.

#### Characterization

Sample morphologies were characterized by an AMRAY 1000B scanning electron microscope (SEM). The phase compositions of the samples were identified through X-ray diffraction (XRD) by Rigaku D/max-A diffractometer with Co Kα radiation. The FTIR spectra of the sample was investigated via a Fourier transform infrared spectroscope (FTIR, Themo Nicolet 670FT-IR). The microstructural characteristics of samples were observed by high-resolution transmission electron microscope (JEM-2010), and the selected area electron diffraction (SAED) technique was used to identify the lattice structure of the samples. Specific surface areas were determined by the Brunauer-Emmet-Teller analysis in the Nitrogen adsorption-desorption measurements at 77 K using Micromeritics Tristar apparatus. For electronic conductivity measurements, the powders were pressed into pellets and sintered at 400 °C under argon atmosphere for 12 h, and the electrical conductivity was measured by a four-electrode method using a conductivity detection meter (Shanghai Fortune Instrument Co., Ltd, FZ-2010).

#### Electrochemical Measurements

Half-cell studies were performed to conduct electrochemical performance evaluation. During the preparation of experimental electrode, we use PVDF and acetylene black powder to serve as conductive binder and additive, respectively. The synthesized TiO_2_@SnO_2_ nanocapsules were mixed with acetylene black and PVDF, and then dissolved in N-methyl-pyrrolidinone with the weight ratio of 80:10:10. This slurry was then spread onto a copper foil used as current collector. After removing the solvent by evaporation, the electrode dried at 120 °C under vacuum for 72 hours. Metallic lithium foil was used as counter electrode. The test cell was assembled in argon filled glove-box. The electrolyte was 1 M LiPF_6_ in a mixture 1:1 molar ratio of dimethyl carbonate (DMC) and ethylene carbonate (EC). The lithium foil was used as the counter electrode. Galvanostatic charge-discharge test was used to study the cyclic performance at needed current density by Land 2100 A tester, in a voltage range of 0.01–3.0 V versus Li/Li^ + ^. Cyclic voltammetry (CV) test was used to detect the electrochemical kinetics during charge and discharge process between 0.01 and 3.0 V with a scan rate of 0.01 mV s^−1^.

## Results and Discussion

### Structure and morphology of hierarchical hollow SnO_2_@TiO_2_ nanocapsules

**Figure 2:**
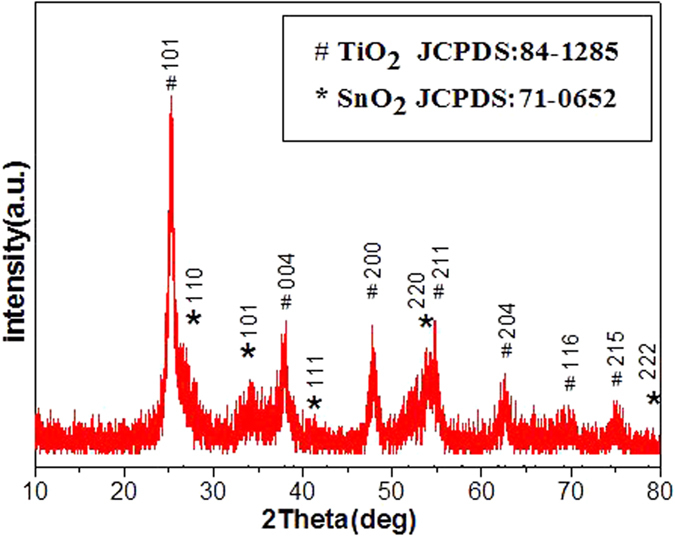
XRD pattern of hollow SnO_2_@TiO_2_ (a) and its precursor of SnO_2_ (b).

**Figure 3:**
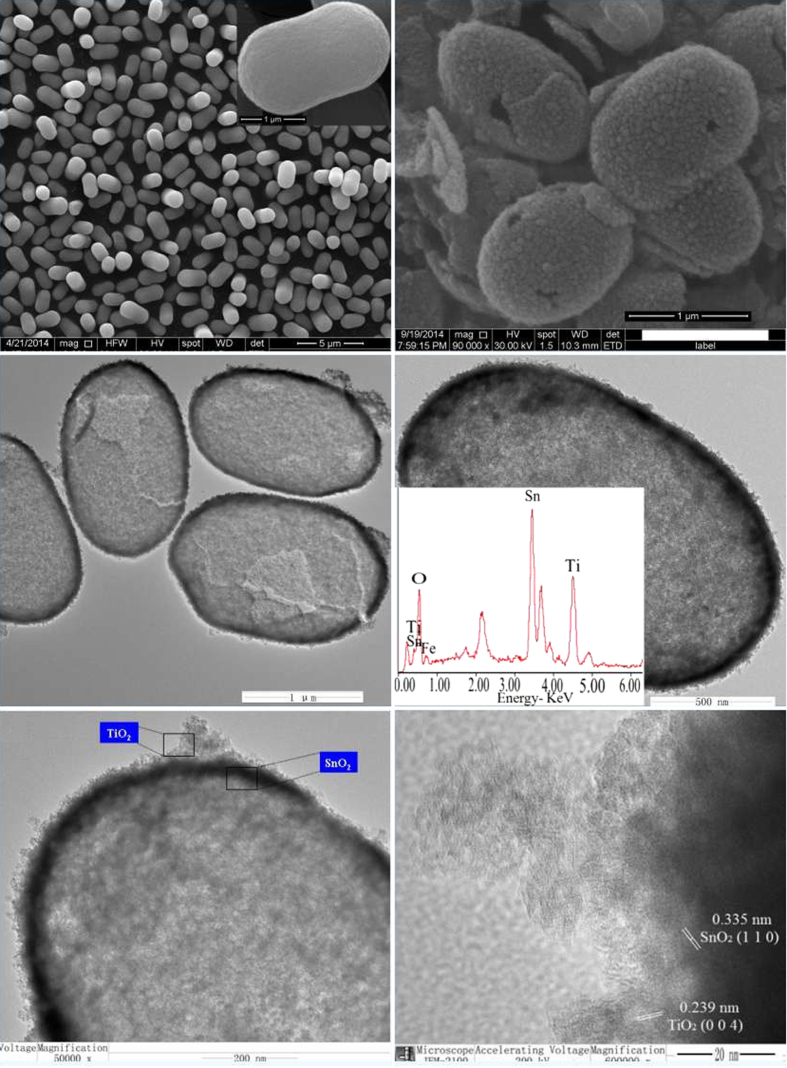
SEM images of monodisperse α-Fe_2_O_3_ particles with capsule shapes (**a**).SEM image (**b**), TEM images (**c**–**e**), and HRTEM micrographs (**f**) of as-synthesized hollow SnO_2_@TiO_2_ nanocapsules. The inset in (**d**) shows EDX analysis.

**Figure 4:**
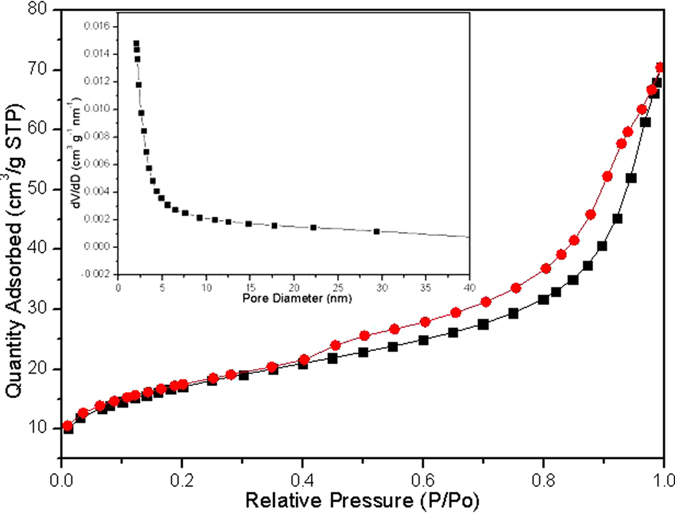
Nitrogen adsorption-desorption isotherm and Barrett-Joyner-Halenda (BJH) pore size distribution plot (inset) of the prepared hollow SnO_2_@TiO_2_ nanocapsules.

The crystallographic structure of the precursors and the as-synthesized final products are analyzed by XRD, as shown in Fig. 2. The precursor can be indexed to a tetragonal rutile SnO_2_ structure (JCPDS card No. 41–1445) without any other impurities. The as-prepared final samples are assigned to SnO_2_ coupled with tetragonal anatase TiO_2_ structure (JCPDS card No. 21–1272). The crystallite size is calculated ca. 2–5 nm using the Scherrer equation, according to the peak broadening of the (1 0 1) reflection of TiO_2_ and (1 1 0) of SnO_2_ reflection, implying the particles are composed of nanocrystal subunits. The SEM images of the prepared α-Fe_2_O_3_ capsule particles and hollow SnO_2_@TiO_2_ nanocapsules yielded by calcinations at 450 °C at different magnifications are shown in Fig. 3a,b. It is obvious that the synthesized SnO_2_@TiO_2_ samples maintain the similar morphology as that of prepared α-Fe_2_O_3_ except for a little shrinkage in size. It can be seen that the particles have a hollow structure evidenced by the partially broken shell with a shell thickness of ca. 30–50 nm as shown in Fig. 3b. The surface of the synthesized particles is made up from nano-sized particles, which might be induced by the rapid mass-transport across the shells during the fast dissolution of α-Fe_2_O_3_. The unique capsule morphology of SnO_2_@TiO_2_ can also be characterized by both TEM and HR-TEM, as illustrated in Fig. 3c–f. The low-magnification TEM image in Fig. 3c shows clearly a visible hollow interior structure. In particular, a typical nanocapsule with well-defined interior and very thin shell can be seen by Fig. 3d, which is in a well agreement with SEM analysis. The thickness of shell of the nanocubes is ca. 50 nm. From Fig. 3e, it can be seen that the surface of SnO_2_ is coated by a thin film of TiO_2_. The HRTEM micrograph (Fig. 3f) for a small location of the particle further shows that the lattice spacings are 0.335 nm and 0.239 nm, corresponding to (1 1 0) plane spacing of SnO_2_ and (0 0 4) plane spacing of TiO_2_ in a spinel structure, respectively. These results are in well agreement with what we have observed from both XRD and EDX analyses. The isotherms of N_2_ adsorption/desorption and the pore size distribution of the obtained hierarchical hollow SnO_2_@TiO_2_ nanocapsules are shown in Fig. 4. The characteristic isotherm is the type of mesoporous materials. The average pore diameters of the sample ranged from 4.5 to 7.8 nm, according to the pore size distribution data. The BET surface area of the SnO_2_@TiO_2_ nanocapsules is 72.12 m^2^ g^−1^. The single-point total volume of pores is 0.241 cm^3^ g^−1^ (P/P_0_ = 0.975). These results indicate that the fabricated products are a loose mesoporous structure. It is believed that this structure can not only keep the nano-effect of active components but also help to buffer the volume changes of the electrode during electrochemical cycling.

### Synthesis mechanism of nanocapsules with controlled morphologies

**Figure 5:**
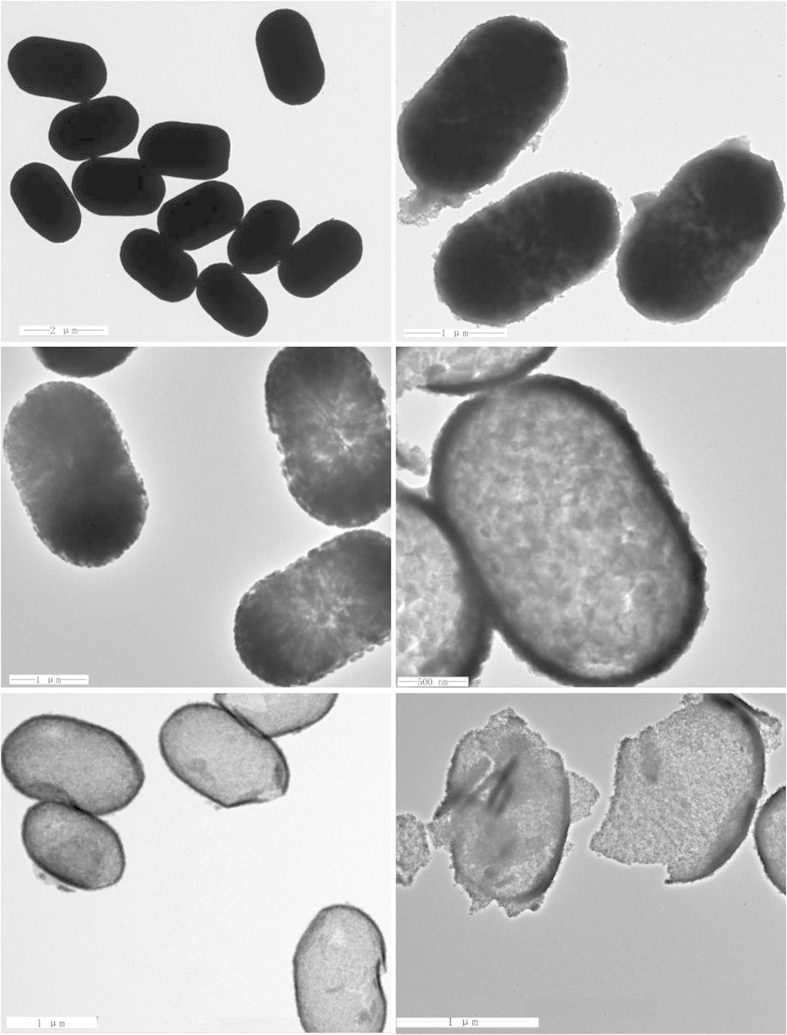
TEM images of hollow SnO_2_ nanocapsules obtained by etching of solid α-Fe_2_O_3_ particles at 100 °C for 0 min (a), 10 min (b), 20 min (c), 30 min (d), 40 min, and 50 min (f).

Regarding the formation mechanism of hollow TiO_2_@SnO_2_ nanocapsule, the formation mechanism of SnO_2_ may be adopted for analysis. Figure 5 shows the TEM images of microwave-assisted HCl etching of α-Fe_2_O_3_ capsule at 100 °C at different etching time periods from 0 to 50 minutes. The formation process is from the particle center to outside under ambient conditions. Figure 5a shows the starting particles. When the reactions are conducted for 10 minutes (Fig. 5b) and 20 minutes (Fig. 5c), the surface of sample becomes a little rough and is transformed into hollow ones gradually. With increasing the reaction time up to 30 minutes, the hollow capsule structures become more dominating (Fig. 5d). Further prolonging the reaction time, for example, to 40 minutes, the interior of the nanocubes becomes empty (Fig. 5e). Finally, the hollow crossed nanocubes start to be broke when the reaction time is additionally high up to 50 minutes (Fig. 5f). In this case, it is believed that the reaction runs much fast, leading to a different dynamic process from the traditional concept of the sacrificial-template. Compared with the procedures reported in literature, our strategy can provide a novel route for preparing hollow capsule-structured materials with shorter time, much larger quantity, and lower cost. This route can also be used to prepare other cage-bell structured materials, such as TiO_2_ (Figure S1), CeO_2_ (Figure S2) and NiO (Figure S3), for details see supporting information.

### Electrochemical characterizations of hierarchical hollow SnO_2_@TiO_2_ nanocapsules

**Figure 6:**
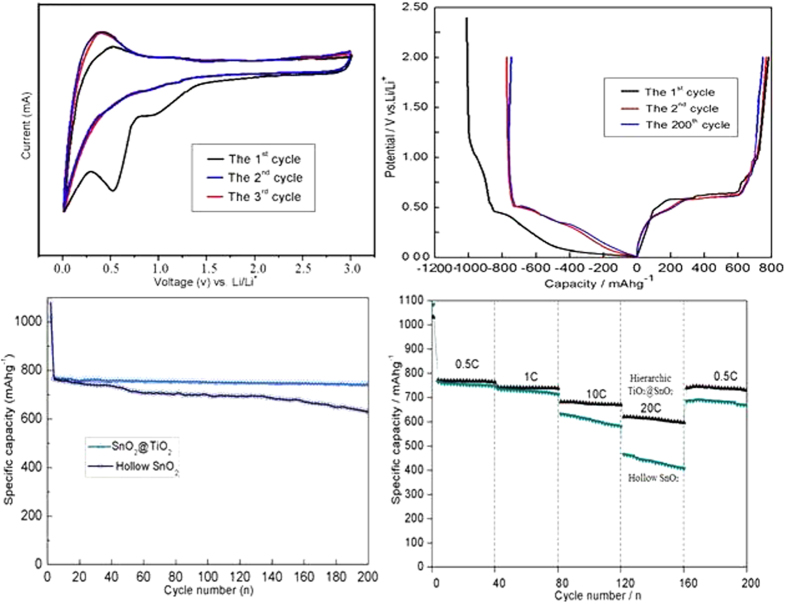
Electrochemical performances of as-prepared hierarchical hollow nanocapsules (etching time of 40 min): (**a**) cyclic voltammetry plots of SnO_2_@TiO_2_ electrode at the scan rate of 0.05 mV s^−1^. (**b**) charge/discharge curves of SnO_2_@TiO_2_ electrode for the 1^st^, 2^nd^, and 200^th^ cycle at current density of 1 C; (**c**) the cycling performance of bare SnO_2_ and SnO_2_@TiO_2_ measured at 1 C. (**d**) rate capability of SnO_2_@TiO_2_ electrode from 0.5 C to 20 C for 200 cycles. Electrode potential range; 0.01–3.0 V vs. Li/Li^+^.

The packing density and conductivity of the obtained hierarchical hollow SnO_2_@TiO_2_ nanocapsules, synthesized by microwave-assisted etching of Fe_2_O_3_ template at 100 °C for 40 minutes and subsequent calcinations in air at 450 °C, are measured to be 1.32 g cm^−3^ and 9.85 × 10^−3^ Ω^−1^cm^−1^, respectively. While the conductivity of commercial SnO_2_ is only ca. 1.055 × 10^−8^ Ω^−1^cm^−1^, demonstrating the significantly improved electron transport due to TiO_2_ coating. To test the material’s electrochemical performance, the prepared hollow TiO_2_@SnO_2_ nanocapsules were used to fabricate the anode for LIBs, as described in the experimental section. From Fig. 6a, it can be clearly seen that the SnO_2_@TiO_2_ electrode shows a very stable reversible capacity of 772 mAh g^−1^, which can be retained at 742 mAh g^−1^ after 200 cycles with the retention of 96.1%. The coulombic efficiencies are always over 99.5% except for the first cycle at 1 C (1 C = 780 mAh g^−1^). However, the bare SnO_2_ sample presents only 628 mAh g^−1^ under the same measuring conditions. Obviously, the SnO_2_@TiO_2_ exhibits a highly improved reversibility due to the coupling effect between TiO_2_ and SnO_2_. Figure 6b shows the charge/discharge curves of hierarchical hollow SnO_2_@TiO_2_ nanocapsule (etching time of 40 minutes) based electrode for the first two and the 200^th^ cycle at a constant current density of 1 C. It can be seen that in the initial discharge, the potential drops rapidly to a plateau of 0.85 V and then decreases gradually to the plateaus of approximately 0.61 and 0.35 V, respectively. The reasons might be ascribed to the formation of solid electrolyte interface (SEI) film, that is, Li_2_O and Li_x_Sn (0 ≤ x ≤ 4.4) alloy, as described in literature^31^,^32^,^33^. From the second cycle, the initial potential plateau increases from 0.3 V to ca. 0.4 V. After 200 cycles, the capacity can still be kept at ca.740 mAh g^−1^, suggesting the excellent reversibility of electrode. Figure 6c shows the cyclic voltammograms (CVs) on the TiO_2_@SnO_2_ nanocapsule electrode within the range of 0.01–3.0 V vs. Li/Li^+^, where two pairs of redox peaks can be observed. The first pair of current peaks at around (cathodic/anodic) 0.82/1.26 V (the cathodic peak appears at a lower voltage range in the initial cycle) can be attributed to the partially reversible conversion between SnO_2_ and Sn, while the more pronounced pair at a lower potential of 0.01/0.65 V should correspond to the alloying/de-alloying processes that contribute to the major part of the capacity. These results are consistent with CV analysis above and other reports^4^,^34^.

**Figure 7:**
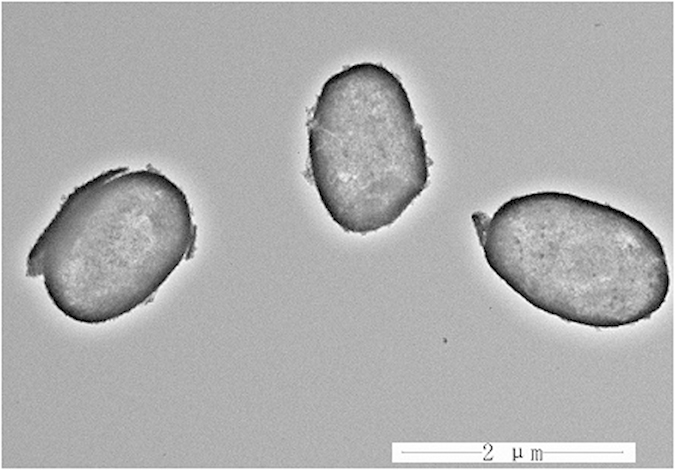
TEM image of hybrid hollow SnO_2_@TiO_2_ nanocapsules electrodes after 200 cycles at 1 C.

To investigate electrochemistry performance under different rates of discharge, Fig. 6d presents the discharge capacities of hierarchical hollow TiO_2_@SnO_2_ nanocapsules and bare SnO_2_ electrodes as a function of current rates from 0.5 C to 20 C, and each sustained for 40 cycles. It can be seen that the stable cyclic performance of TiO_2_@SnO_2_ nanocapsule-based electrode can be achieved for all rates. A specific capacity of ca. 730 mAh g^−1^ is recovered when the current rate is reduced back to 0.5 C after 200 cycles at higher rates. It should be mentioned that the rate performance of TiO_2_@SnO_2_ electrode is much higher than that of pure SnO_2_ one, and also much better than other SnO_2_-based materials at such high rates in literature^4^,^32^,^33^,^34^. According to the TEM morphologies of SnO_2_@TiO_2_ (Fig. 7) and pure SnO_2_ (Figure S4, seeing the supporting information) electrodes after 200 cycles at a current density of 1 C, the prepared nanocapsule structure can still be retained without any breakage in the process of charge-discharge. This implies that the nanocapsule electrodes synthesized in this work process a significantly improved durability. Therefore, the synthesized SnO_2_@TiO_2_ nanocapsules can exhibit the significantly enhanced electrochemical performance when compared with the previous reported SnO_2_-based materials^4^,^32^,^33^,^34^,^35^,^36^.

## Conclusions

Hierarchical hollow SnO_2_@TiO_2_ nanocapsules are successfully synthesized by a simple and fast benign procedure combining with microwave-assisted HCl etching reaction and subsequent calcinations. The thickness of TiO_2_@SnO_2_ nanocapsule shell is measured to be ca. 40 nm. When this noval material is used to fabricate the anode for LIBs, a stably reversible capacity of 770 mAh g^−1^ can be achieved and can be retained at 740 mAhg^−1^ even after 200 cycles with the retention of 96.1%. Based on the results described in this paper, the following conclusions can be reached: (1) in hierarchical hollow TiO_2_@SnO_2_ nanocapsules, the unique hollow nanocages can shorten the length of Li-ion diffusion, which is benefit for the rate performances; (2) the hollow structure can offer a sufficient void space, which can sufficiently alleviate the mechanical stress caused by volume change; (3) the hybrid elements allow the volume change to take place in a stepwise manner during the electrochemical cycling; and (4) the data of overall rate performance confirm again the importance of TiO_2_-coating toward high capacities in both low and high current rates. Due to these advantages, the hierarchical hollow TiO_2_@SnO_2_ nanocapsule electrode can exhibit an extraordinary electrochemical performance. In addition, The synthesis strategy presented in this work is simple, cheap and mass-productive, which may shed light on a new avenue for the fast synthesis of hierarchically hollow structural nano/micro-functional materials for energy storage, catalyst, sensor, and other new applications.

## Additional Information

**How to cite this article**: Zhou, L. *et al.* Morphology-controlled construction of hierarchical hollow hybrid SnO_2_@TiO_2_ nanocapsules with outstanding lithium storage. *Sci. Rep.* **5**, 15252; doi: 10.1038/srep15252 (2015).

## Supplementary Material

Supplementary Information

## Figures and Tables

**Figure 1:**
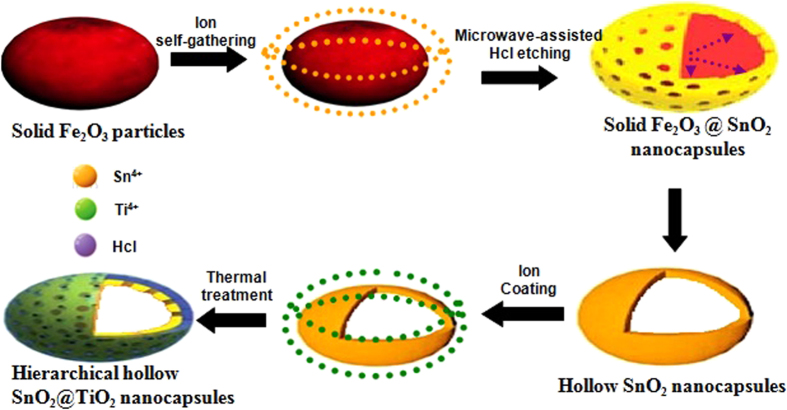
Representative illustration of the formation of hierarchical hollow SnO_2_@TiO_2_ nanocapsules by the microwave-assisted etching of Fe_2_O_3_ template. (This figure is drawn by L.X.L.).
