# Supplementary material for: Morphology-controlled construction of hierarchical hollow hybrid SnO2@TiO2 nanocapsules with outstanding lithium storage
Source: Sci Rep. 2015 Oct 20;5:15252. doi: 10.1038/srep15252 (PMC4611182; doi:10.1038/srep15252)
Supplement: Supplementary Information [file srep15252-s1.doc]

**Supporting Information**

**Morphology-controlled construction of hierarchically hollow hybrid SnO_2_@TiO_2_ nanocapsules with outstanding lithium storage**

Linzong Zhou^a,b^, Hong Guo^a^*, Tingting Li^a^ , Weiwei Chen^a^, Lixiang Liu^a^, Jinli Qiao^c*^ and Jiujun Zhang^d^

a. School of Chemistry Science and Engineering, Yunnan University, Kunming 650091,Yunnan, China

b. School of geographical science and tourism management, Chuxiong Normal University, Chuxiong 675000, Yunnan, China

c. College of Environmental Science and Engineering, Donghua University, Shanghai 201620, China

d. Department of Chemical Engineering, E6-2006, University of Waterloo, Waterloo, ON, N2L 3G1, Canada

*Corresponding author email address: a. [guohongcom@126.com](mailto:guohongcom@126.com); c. [qiaojl@dhu.edu.cn](mailto:qiaojl@dhu.edu.cn);

**
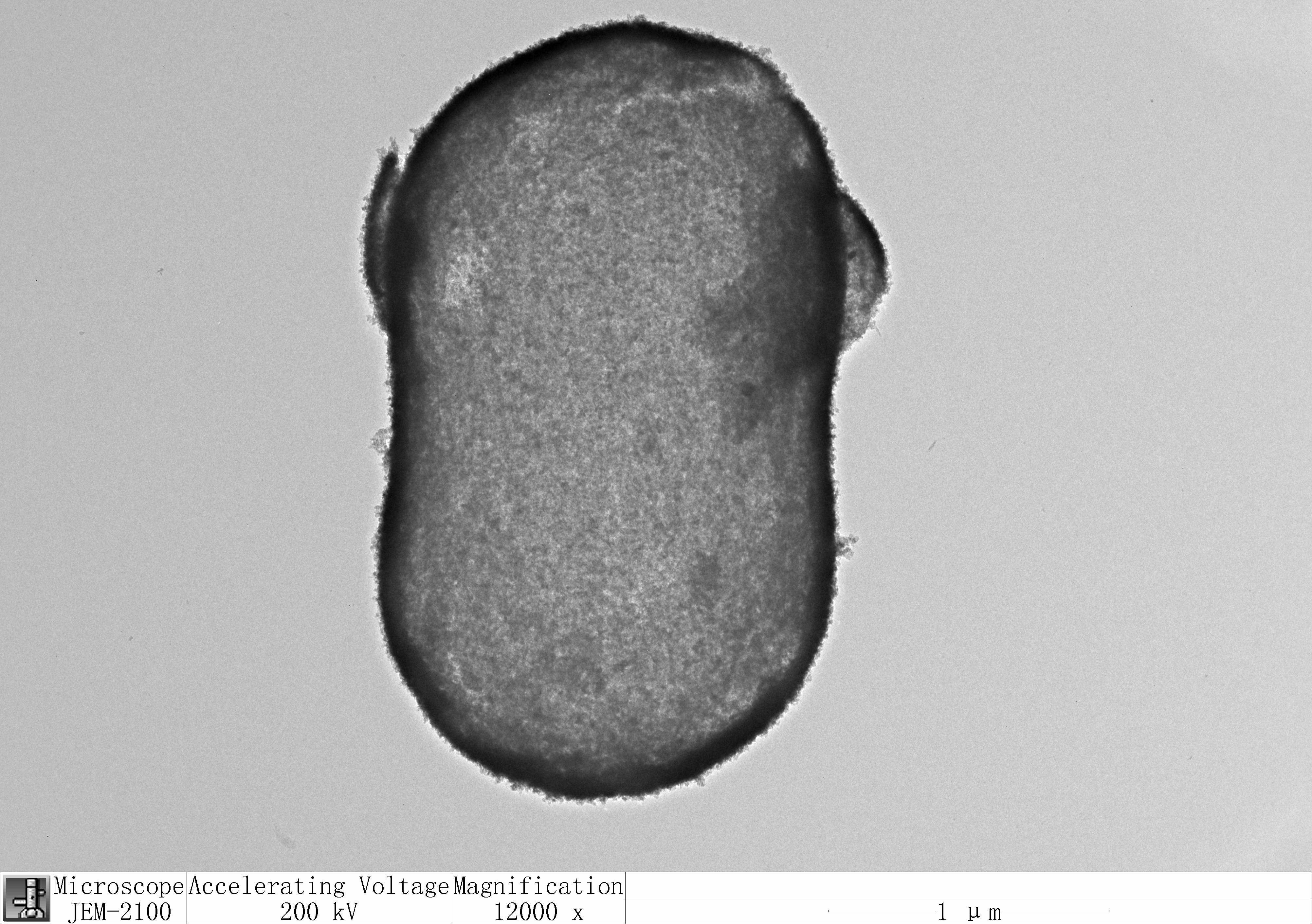
**

**Fig. S1** TEM image of the obtained hollow TiO_2_ capsules.


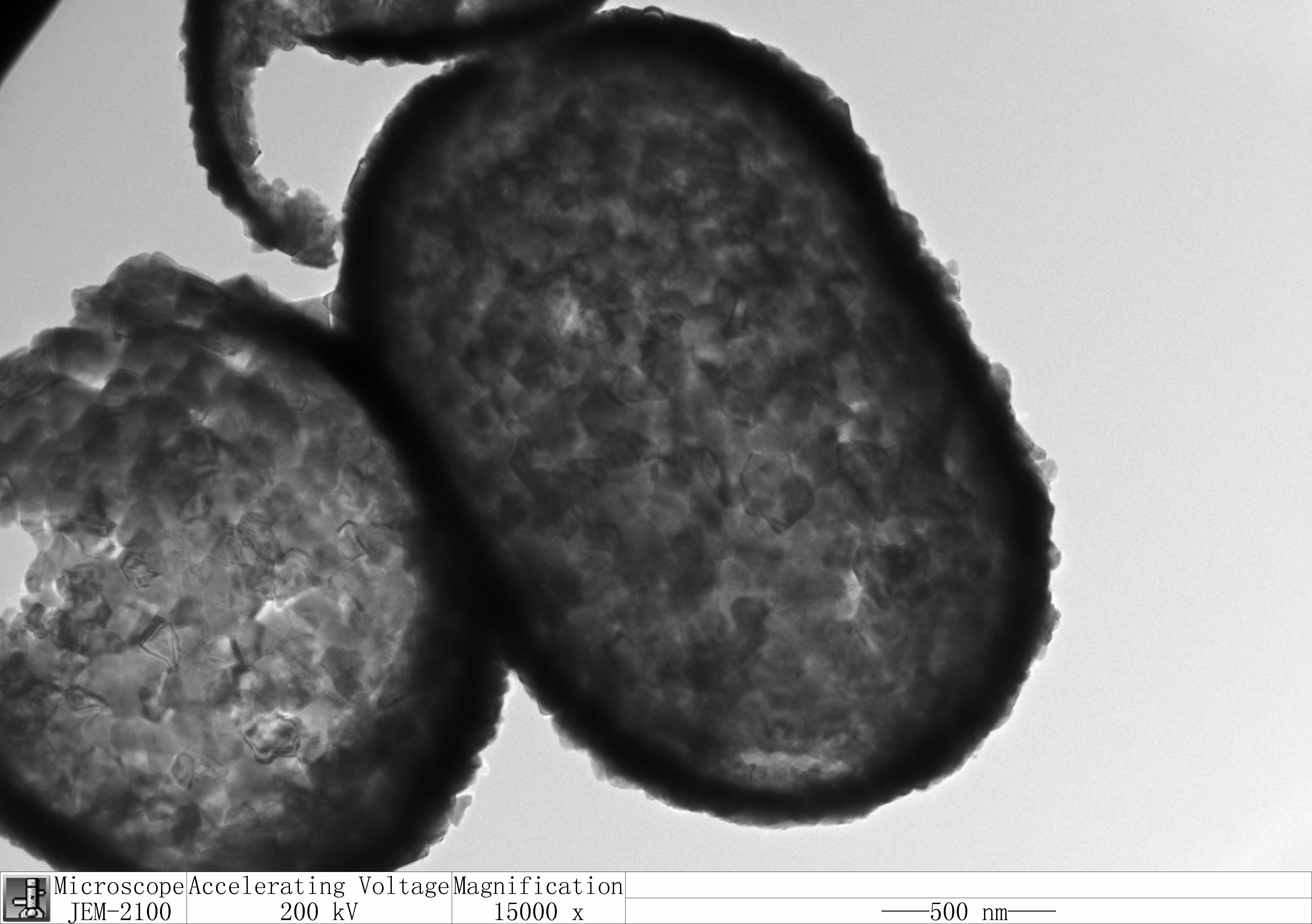


**Fig. S2** TEM image of the obtained hollow CeO_2_ capsules.


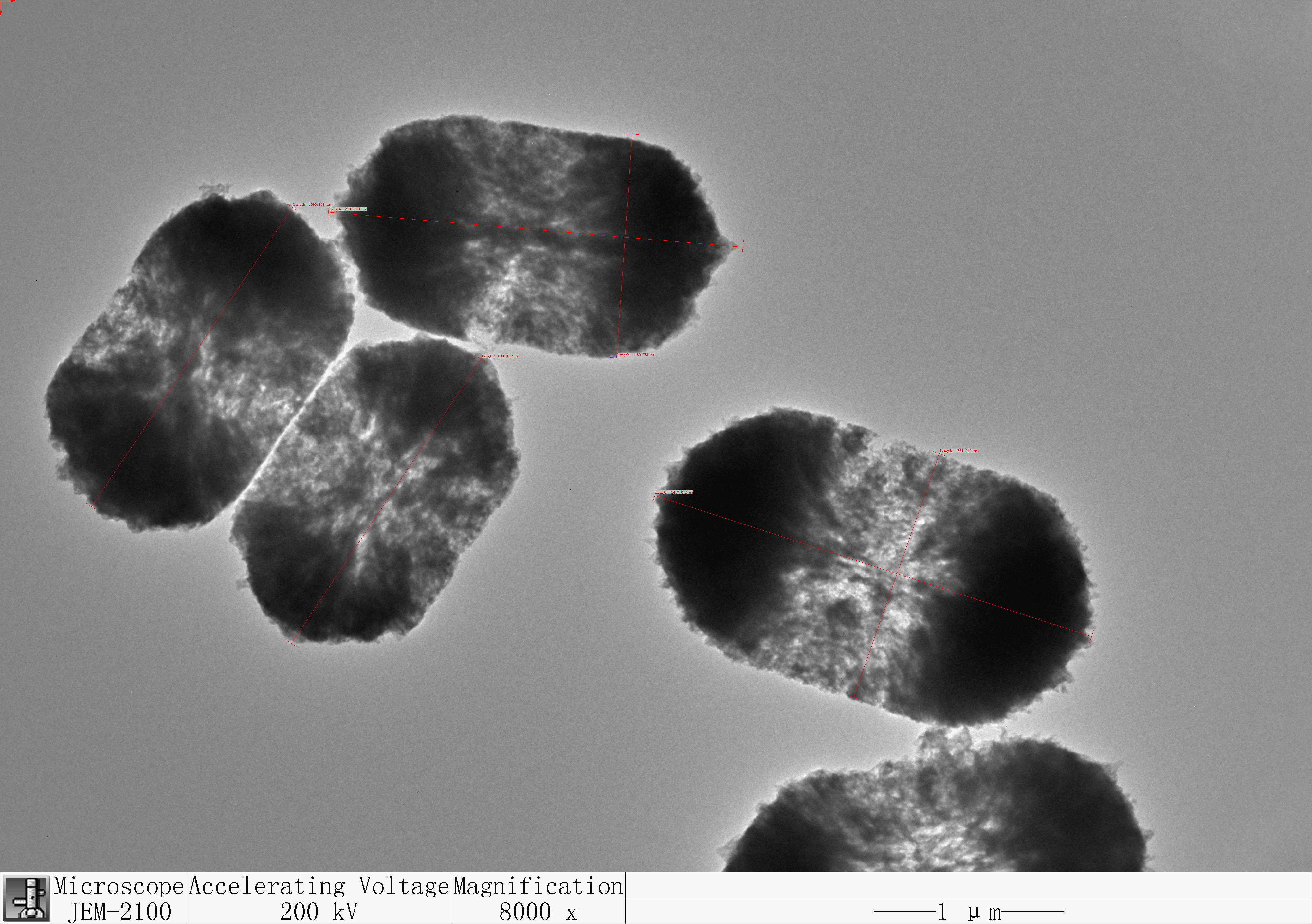


**Fig. S3** TEM image of the obtained hollow NiO capsules.

**
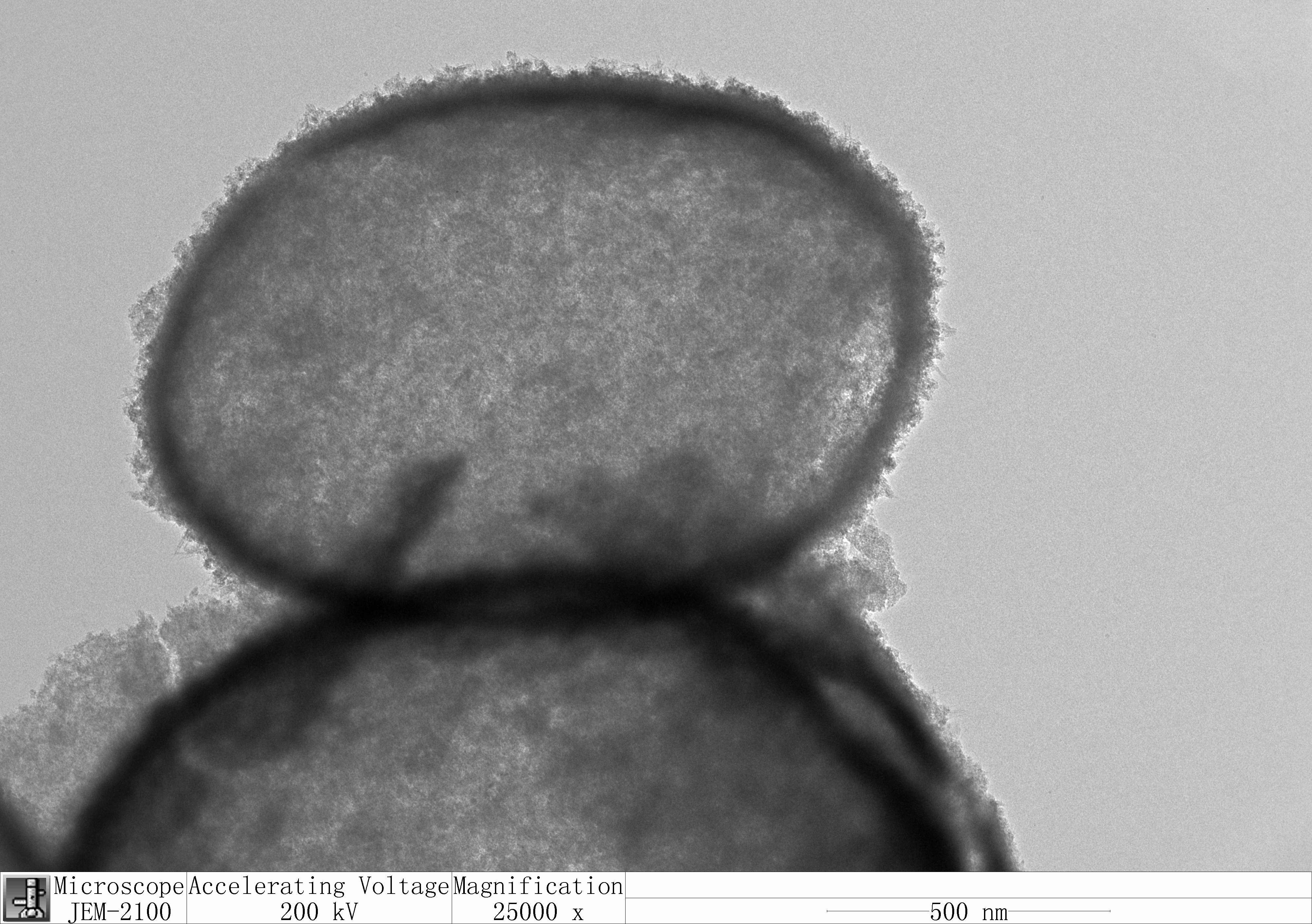
**

**Fig. S4** TEM image of hollow SnO_2_ electrodes after 200 cycles at 1C.
